# The Role of Dietary Fats in the Development and Prevention of Necrotizing Enterocolitis

**DOI:** 10.3390/nu14010145

**Published:** 2021-12-29

**Authors:** Belal N. Alshaikh, Adriana Reyes Loredo, Megan Knauff, Sarfaraz Momin, Shirin Moossavi

**Affiliations:** 1 Neonatal Nutrition and Gastroenterology Program, Department of Pediatrics, Cumming School of Medicine, University of Calgary, Calgary, AB T2N 2T9, Canada; Adriana.ReyesLoredo@albertahealthservices.ca (A.R.L.); Sarfaraz.Momin@albertahealthservices.ca (S.M.); 2 Nutrition Services, Alberta Health Services, Calgary, AB T2N 2T9, Canada; Megan.Knauff@albertahealthservices.ca; 3 Department of Physiology and Pharmacology, Cumming School of Medicine, University of Calgary, Calgary, AB T2N 2T9, Canada; shirin.moossavi@ucalgary.ca; 4 International Microbiome Centre, Cumming School of Medicine, Health Sciences Centre, University of Calgary, Calgary, AB T2N 2T9, Canada

**Keywords:** fatty acids, necrotizing enterocolitis, intestinal inflammation, preterm infants

## Abstract

Necrotizing enterocolitis (NEC) is a significant cause of mortality and morbidity in preterm infants. The pathogenesis of NEC is not completely understood; however, intestinal immaturity and excessive immunoreactivity of intestinal mucosa to intraluminal microbes and nutrients appear to have critical roles. Dietary fats are not only the main source of energy for preterm infants, but also exert potent effects on intestinal development, intestinal microbial colonization, immune function, and inflammatory response. Preterm infants have a relatively low capacity to digest and absorb triglyceride fat. Fat may thereby accumulate in the ileum and contribute to the development of NEC by inducing oxidative stress and inflammation. Some fat components, such as long-chain polyunsaturated fatty acids (LC-PUFAs), also exert immunomodulatory roles during the early postnatal period when the immune system is rapidly developing. LC-PUFAs may have the ability to modulate the inflammatory process of NEC, particularly when the balance between n3 and n6 LC-PUFAs derivatives is maintained. Supplementation with n3 LC-PUFAs alone may have limited effect on NEC prevention. In this review, we describe how various fatty acids play different roles in the pathogenesis of NEC in preterm infants.

## 1. Introduction

Necrotizing enterocolitis (NEC) is the most common gastrointestinal emergency in preterm infants and is a major cause of mortality and morbidity in the neonatal intensive care unit (NICU). A systematic review and meta-analysis indicated that confirmed NEC is associated with 23% mortality, 24–61% neurodevelopmental disability, and 15–35% intestinal failure [1]. The pathogenesis of NEC is multifactorial and not completely understood. Intestinal immaturity, abnormal microbial colonization, ischemic-reperfusion injury, and highly immunoreactive intestinal mucosa are all thought to be important factors that may lead to NEC [2]. NEC occurs mostly in infants on enteral feeds. Feeding human milk, either the mother’s own milk or donor human milk, remains the most protective measure against NEC. Several studies including randomized trials have shown that the incidence of NEC is 2- to 10-fold lower in human milk-fed infants compared with formula-fed infants [3]. Formula-fed infants are typically at the highest risk for NEC; therefore, several formula components including fat are suggested to play a critical role in the disease development [4]. In contrast, feeding breastmilk has often been shown not only to prevent NEC, but also to promote gastrointestinal health [5].

Preterm infants have higher energy needs than their term counterparts in the early postnatal life [6,7]. Fat provides approximately 50% of the preterm infant’s caloric needs and supports many physiologic and metabolic functions that are vital to their growth and neurodevelopment [8,9,10]. Furthermore, several fatty acids play fundamental immunomodulatory roles by regulating key pathways for inflammatory responses [9,11]. Human milk contains long chain polyunsaturated fatty acids (LC-PUFAs), mainly arachidonic acid (AA) and docosahexaenoic acid (DHA), that modulate intracellular signaling within immune cells. A balance between the n6 LC-PUFAs and the n3 LC-PUFAs promotes lipid mediator formation that is crucial to achieving protection against pathogens without exaggerated inflammation [12]. Unlike infant formulas, breastmilk contains a broad fatty acid profile. Unfortified breastmilk is nutritionally inadequate to meet the fat and energy needs of preterm infants. Therefore, supplementation with multinutrient products that contain vegetable and synthetic fats has become the standard practice in this population. Since several fatty acids are strongly involved in preserving intestinal homeostasis and modulating inflammatory responses, the association between fat and NEC cannot be overlooked. The objective of this review is to outline the potential effects of different fatty acids on the development of NEC in preterm infants.

## 2. Postnatal Fatty Acid Status in Preterm Infants

In the third trimester of pregnancy, the fetus increases its nutrient demands to support rapid tissue growth with fat deposition. Preterm birth interrupts the placental transfer of the fatty acids. Parenteral lipid has become the standard of care to meet the early fat requirements of preterm infants after birth. Following the weaning of parenteral lipid, preterm infants rely on enteral sources to meet these requirements, whether through breastmilk and or formula. Since the fat content of the intravenous lipid emulsions (IVLEs) and enteral nutrition is inadequate to meet preterm infants’ needs for more than a few days, postnatal deficits of certain fatty acids accumulate quickly. For example, the DHA status of extremely low birth weight (ELBW) infants declines significantly in the first 2 months after birth, particularly in infants exposed to IVLEs for more than 28 days [13]. This low DHA status can remain for weeks, even after the establishment of full enteral feeding [14]. Several other plasma fatty acids abnormalities such as low (AA) and high linoleic acid (LA) are also described in very preterm infants [15]. Some of these abnormalities are associated with neonatal morbidities. A study by Martin et al. revealed that the deficit in DHA was associated with increased risk of bronchopulmonary dysplasia (BPD), while the decreased AA and high LA levels were associated with increased risk of nosocomial sepsis [15]. The number of infants who developed NEC in the former study was limited to 5, making it difficult to examine the association between plasma fatty acids levels and NEC [15]. Nevertheless, common morbidities in preterm infants often involve elements of uncontrolled inflammation. Current evidence suggests that alterations in LC-PUFA delivery to preterm infants may have negative implications on the risk of neonatal morbidities including NEC [11,16,17].

## 3. Fatty Acids of Breastmilk

Feeding human milk has been shown to improve gastrointestinal function and reduce the incidence of NEC. Term and preterm infants are born with an immature immune response. The maturation of an infant’s immune system is supported by breast milk fatty acids including the LC-PUFAs AA and DHA. Unlike infant formulas, the fatty acid profile of human milk is influenced by maternal diet. Despite maternal diet variability, breastmilk provides a relatively constant n6 to n3n3 ratio. LCPUFAs may contribute to the immune benefits of human breast milk; however, the extent of this contribution is unclear [12].

### 3.1. Fatty Acids Content and Structure

The average human milk fat content is 3.5 g/100 mL with wide variation between 1.8 and 4.9 g/100 mL [6]. This variation depends on many factors such as lactation period, feeding stages (foremilk and hindmilk), and dietary habits of mothers [18,19]. For donor human milk, it is further influenced by processing and pasteurization [20].

**Figure 1:**
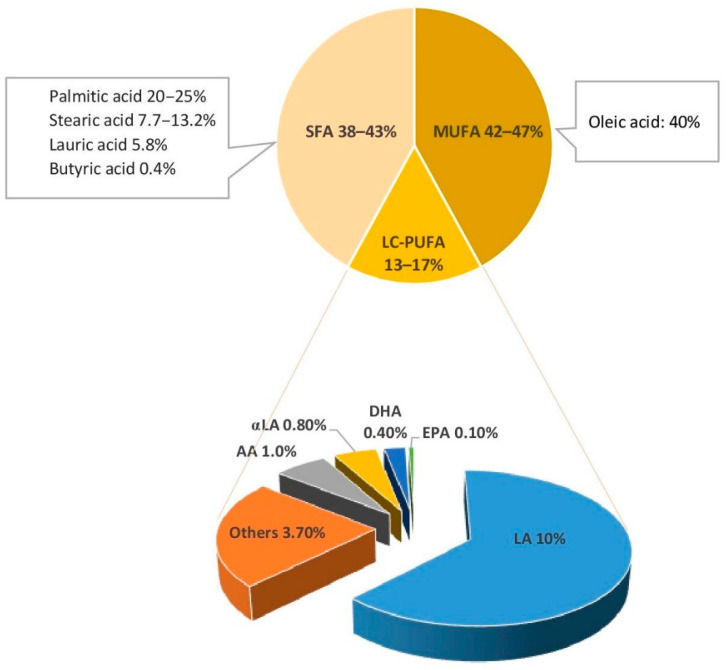
Fatty acids of breastmilk. SFA, saturated fatty acids; MUFA, mono unsaturated fatty acids; LC-PUFA, long chain-poly unsaturated fatty acids; LA, linoleic acid; αLA,α linolenic acid; AA, arachidonic acid; EPA, eicosapentaenoic acid; DHA, docosahexaenoic acid [23,24].

Triglycerides (TGs), which compose 98% of breastmilk fat, consist of two main components: a glycerol backbone and three fatty acids tails [21,22]. The structure of these fatty acids depends on a number of molecular characteristics including the number of carbon atoms, the presence or absence of unsaturated bonds, their number, and their position in the TG molecule [21]. Fatty acids are classified as saturated or unsaturated according to the number of double bonds in the hydrocarbon chain. Monounsaturated fatty acids (MUFAs) have one double bond, whereas polyunsaturated fatty acids (PUFAs) have several. LC-PUFAs (18 or more carbons) comprise 90% of all PUFAs in breastmilk [14]. They are classified into two key families: n3 and n6, depending on the position of the first double bond in the hydrocarbon chain. Breastmilk typically contains 35–40% saturated fatty acids (SFAs), 45–50% MUFAs, and approximately 15% LC-PUFA [23]. Figure 1 shows the type and concentration of fatty acids of breastmilk.

### 3.2. Role of Fatty Acid Balance in the Infant Diet

LC-PUFAs have many structural, energetic, and metabolic functions. LA (n6) and α-linolenic acid (αLA; n3) are PUFAs known as ‘essential fatty acids’ (EFAs). EFAs are converted into metabolites to exert pro- and anti-inflammatory actions. LA is converted to AA (n6), which is the main precursor of thromboxanes, prostaglandins, and leukotrienes. These metabolites are common precursors for the inflammatory response of NEC [25]. Derivatives of αLA include DHA (n3) and eicosapentaenoic acid (EPA; n3). DHA and EPA are precursors of series-3 prostaglandins that inhibit platelet aggregation [26].

Preserving an appropriate ratio between SFAs, MUFAs and LC-PUFAs, and between the n3 and n6 LC-PUFAs derivatives, DHA and AA, is important to maintain intestinal cytokine balance and prevent intestinal necrosis and apoptosis. Animal studies showed that modifying fatty acid composition of the early diet influences intestinal membrane fatty acids, with effects on permeability and inflammatory pathways that persist into later life [27,28]. Suppression of AA (n6; LC-PUFAs) by feeding female rats diets with 20% energy from safflower oil (15% oleic acid; n9 MUFA and 75% LA; n6 LC-PUFA) or canola oil (60% oleic acid; n9 MUFAs and 21% LA; n6 PUFA), 8% fish oil (n3 PUFA) plus 2% soy oil (51% LA and 23% oleic acid), or 18% fish plus 2% soy oil throughout gestation and lactation resulted in significant aberration in the normal trajectory of intestinal development with reduced crypt depth in their pups on a fish oil diet [28]. Moreover, the intestine of pups on the fish oil diets had a long-lasting heightened inflammatory response to experimental colitis in their young adulthood [28]. Another study by Van Greevenbroek et al. showed that supplementation with palmitic acid (SFA) resulted in disturbances in cell morphology and intracellular accumulation of TG precursor molecules in intestinal Caco-2 cells compared to supplementation with oleic acid (MUFAs) and LA (n6 PUFAs) [29,30].

A diet high in SFAs outside of the context of a breastmilk-based diet has been shown to increase metabolic stress and risk of adverse health outcomes [31,32]. Most preterm infant formulas are manufactured to match the fatty acids content of breastmilk and achieve similar balance between SFAs, MUFAs, and LC-PUFAs. However, the absence of breastmilk fat digestive enzymes and the low pancreatic lipase activity in preterm infants are not usually considered when comparing the differences in bioactivity, functions, and intestinal absorption capacity between formula and breastmilk. Recently, a pre-clinical study suggested potential benefits of using predigested fat in formula. [33].

The use of predigested macronutrients to improve the intestinal health of preterm infants is not new. Protein hydrolysate formula has been frequently proposed to improve protein absorption and promote feed tolerance. To date, there is no strong evidence to support the notion that a hydrolyzed protein diet lowers the incidence of NEC or feeding intolerance as it relates to the digestibility and absorption of proteins [34]. There are also no studies that have examined the effect of predigested fat on the incidence or severity of NEC in human preterm infants. In both cases, the lack of evidence to support the benefits of predigested macronutrients warrants further research in this area.

## 4. Fatty Acids in Infant Formula

There are many term formulas and nutrient-enriched preterm formulas available to neonates. Term formula is manufactured to mimic mature human breast milk. The amount of protein, calcium, and phosphate in term formula does not meet the estimated nutrient requirements of preterm infants. In contrast, preterm formula is energy and protein-enriched with higher amounts of vitamins, minerals, and trace elements than term formulas. Preterm formulas are designed to meet intrauterine nutrient accretion rates. The fat content of these formulas is also meant to model that of human milk [35].

Additions of LC-PUFAs to preterm and term formulas have been largely influenced by early reports on positive effects on cognitive development and visual acuity [36,37]. The design of previous studies and the content of current infant formulas may be influenced by these reported benefits. This may have contributed to the lack of strong evidence in NEC.

### 4.1. Fatty Acids Content in Preterm Infant Formulas and Human Milk Fortifiers

As in human milk, the dominant lipids in term and preterm infant formula are TGs. The lipid compositions of infant formulas and human milk fortifiers (HMFs) vary according to the fat sources used to manufacture them. The fat content of most infant formulas is formulated to match the concentration of fat in human milk. Nevertheless, some preterm formulas have 36–43% less fat than donor human milk [38]. HMFs are also designed to match the fat content of breastmilk; however, current evidence suggests that both standard and targeted milk fortification methods lead to fat content that is higher than the recommended intake [39]. It is worth noting that reaching fat content higher than the recommended intake is less likely when using fortified donor human milk.

### 4.2. Source of Fatty Acids in Infant Formula

Vegetable oils are commonly used to provide fatty acids in preterm and term infant formulas and HMFs. The fatty acids found in vegetable oils do not provide adequate amounts of LC-PUFA derivatives, EPA, DHA, and AA [38,40,41]. As a result, most infant formula manufacturers add AA and DHA to their products using fish, algal, or fungal oils [42]. Preterm and term infant formulas contain a high concentration of MUFAs. Oleic acid content has been reported to be higher in some preterm infant formulas than breastmilk [38,43,44].

### 4.3. n3 and n6 LC-PUFAs Balance in Infant Formula

In general, most infant formulas have a wide range of n3 and n6 LC-PUFAs. Given that n3 and n6 PUFAs are competitively metabolized by the same set of desaturation, elongation, and oxygenase enzymes, it is crucial to maintain their balance in infant formula. The European Society for Paediatric Gastroenterology Hepatology and Nutrition (ESPGHAN) recommends the ratio of LA to αLA to be 5:1 to 15:1 [42]. The proposed amount of LA in infant formula is 0.3–1.2 g/100 kcal [42]. When DHA is added to infant formula, it should represent 0.5–1.0% of the total fat content [45].

The addition of LC-PUFAs to preterm formula has theoretical and clinical benefit [46]. LC-PUFAs, particularly the balance between AA and DHA, have important clinical and immunomodulatory roles during the postnatal period when the immune system is rapidly developing. A recent cohort study found that higher mean daily serum levels of DHA during the first 28 postnatal days is associated with less severe retinopathy of prematurity (ROP) even after adjustment for known risk factors. This effect was only seen in preterm infants with sufficiently high AA levels [47]. In this study, the incidence of NEC was three times higher in the infants with severe ROP compared to those with no or mild to moderate ROP [47]. For term infants, formulas enriched in both LC-PUFAs in ratios similar to that of breastmilk have shown to alter immune function markers more comparable to those of exclusively breastfed infants [21,23]. In contrast, feeding infants a diet with high doses of n3 LC-PUFA without additional AA, reduces the n6:n3 LC-PUFA ratio and results in immunosuppressive and anti-inflammatory effects through the reduction in the cell content of AA. [21]. This unbalanced diet is undesirable in the early postnatal period, particularly in preterm infants when the immune system is rapidly developing. Similar findings of the benefits of an optimal ratio between DHA and AA are observed in animal models undergoing experimental NEC. Caplan et al. revealed that the combination of AA and DHA in neonatal rats attenuates the degree of experimental NEC by reducing intestinal inflammation [48]. DHA alone was unable to show any beneficial effect in terms of reducing NEC or Toll-Like Receptor (TLR) 4 expression [48].

## 5. Fatty Acid Digestion and Necrotizing Enterocolitis

### 5.1. Lipase Activity in Preterm Infants

Fat digestion is a complex process. Several lipase enzymes exist throughout the gastrointestinal tract to facilitate the hydrolysis of TGs [49]. Each lipase has a specific affinity and function that is largely based on the site of secretion. Although lingual and gastric lipases account for up to 30% of lipid digestion in orally fed preterm infants, most fat digestion relies on pancreatic and salt-stimulated lipases [50,51,52,53,54]. Studies indicate that preterm infants are deficient in pancreatic lipases, particularly the bile salt-dependent lipase [33,55,56]. Bile salt-dependent lipase is secreted by the lactating mammary gland and allows breastmilk-fed infants to digest fat despite an endogenous deficiency of pancreatic lipase. Therefore, fat digestion and absorption are significantly decreased in formula-fed infants. The addition of recombinant human bile salt-dependent lipase to formula has been shown to improve fat digestion and absorption in preterm infants [57].

### 5.2. Fat Malabsorption and Severity of Necrotizing Enterocolitis

The presence of lipase in the breastmilk supports a rationale for using pre-digested fat in formulas to bypass the requirement for bile salt-dependent lipase for fat digestion and absorption and to potentially prevent NEC in preterm infants. Sodhi et al. induced NEC in neonatal mice using three formulas that contained identical ingredients except for the fat composition. These formulas contained a “standard fat” (long chain TGs in form of unsaturated fatty acids), “reduced TG” (predigested fat), and “very low fat” [33]. The standard fat formula led to severe NEC while using predigested fat and very low fat formulas prevented severe NEC [33]. The increase in mal-digested fat in the mice fed the standard fat formula resulted in a dramatic accumulation of fat droplets within the intestinal epithelium of the distal ileum causing significant generation of reactive oxygen species and intestinal inflammation [33]. These findings suggest a possible role of using hydrolyzed or pre-digested fats to reduce the incidence of severe NEC in preterm infants [33]. It is important to note that this evidence remains preclinical. There is a need for clinical studies in preterm infants prior to implementing such a strategy to reduce the severity of NEC. Clinical studies to reduce NEC is challenging given the need for large sample size to achieve statistical power.

### 5.3. Medium Chain Triglycerides

Formula-fed preterm infants have lower fatty acid absorption capacity as a function of increasing carbon length [58]. Infant formulas include fat in the form of medium chain triglycerides (MCTs) and long chain triglycerides (LCTs) in varying proportions. The absorption of LCTs requires pancreatic lipase, mixed micelles, chylomicrons, and carnitine. The hydrolysis and absorption of LCTs is limited in early life and depends mainly on lingual and gastric lipases [21,23]. The shorter chain lengths of MCTs do not require chylomicron formation and therefore transfer directly to the liver via serum albumin and portal circulation [59]. This allows for faster utilization through direct absorption into the serum without micelles [21,23]. MCT content of preterm and transitional formulas is 40–50% and 20–50%, respectively [60]. Despite the absorption benefits, current evidence suggests limited effects on growth, feeding tolerance, and the risk for developing NEC [61]. Two small randomized clinical trials (RCTs) found no difference in the incidence of NEC between high MCT formulas, containing 30% or more by weight of fat as MCT, and low MCT formula, containing less than 30% of fat as MCT [11,61,62,63]. These two RCTs had small sample sizes and lack the power to detect the difference in the incidence of NEC. The need for large sample size is a challenge for NEC studies in preterm infants given factors inherent to this population.

### 5.4. Stereospecific Positioning of Fatty Acids

The structural component and the orientation of the double bonds of the milk TG molecules is critical for fat absorption [64,65]. Human milk has a unique stereospecific positioning of fatty acids with TG structures enriched in SFAs, particularly palmitic acid (16:0), at the sn-2 (center; sn2-palmitate) position and unsaturated fatty acids at the sn-1 and sn-3 (outer) positions on the glycerol backbone [66]. Pancreatic lipase digestion of TGs with palmitic acid in the sn-2 position maximizes palmitic acid and calcium absorption in addition to overall fat absorption [67]. In contrast, the digestion of palmitic acid in the sn-1 and sn-3 positions results in free palmitic acids that bind with calcium in the intestinal lumen to form insoluble calcium-fatty acids soap complexes that lead to reduced absorption of fatty acids and calcium [68,69]. Most new infant formulas have adequate amounts of palmitic acid from palm olein, but most of these are in the sn-1 and sn-3 position. The use of infant formula with high sn-2 palmitic acid decreases hard stools and crying time, improves calcium absorption, and may promote intestinal health and development [70,71]. In a study by Lu et al. examining the effect of sn-2 versus sn-1,3 palmitic acid on spontaneous enterocolitis, feeding a diet high in sn-2 TGs prevented intestinal mucosal damage and protected against intestinal inflammation through immunosuppressive regulatory T cell responses and upregulation of antioxidant defenses [72]. sn-2 palmitic acid also has favorable impact on the developing intestinal microbiome. In a small randomized trial, term infants fed sn-2 palmitic acid had higher *Lactobacillus* and bifidobacteria compared with those fed palm oil-based formula sn-1,3 palmitic acid, which mimics the association between sn-2 palmitic acid and the favorable intestinal microbiome in infants fed breastmilk [73,74].

## 6. Fatty Acids and Pathogenesis of Necrotizing Enterocolitis

**Figure 2:**
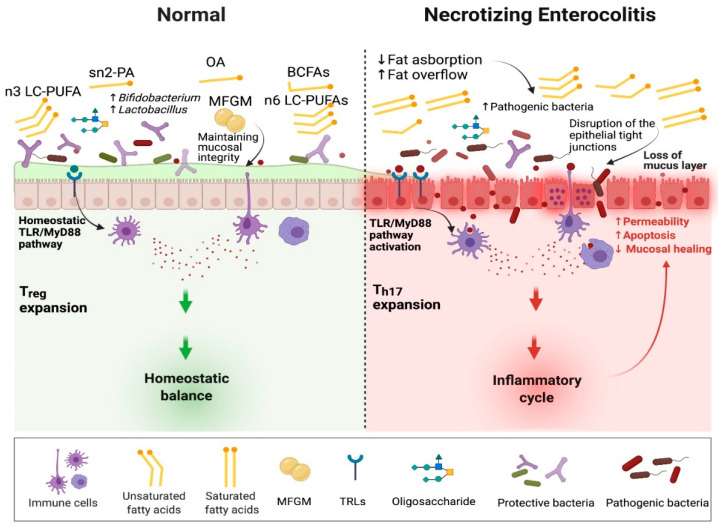
The role of dietary fatty acids in the pathogenesis of necrotizing enterocolitis. Sn2-PA, sn2 palmitic acid; OA, oleic acid; MFGM, milk fat membrane; BCFAs, branched-chain fatty acids; LC-PUFAs, long chain polyunsaturated fatty acids; TLR/MyD88, toll-like receptor/myeloid differentiation primary response gene 88; T_reg_, regulatory T cells; T_h17_, T helper 17 cells.

NEC is typically developed in the setting of disrupted gut microbial colonization and often after administration of non-breastmilk feeds. Increased reactivity of the innate immune system of the intestinal mucosa to microbial ligands and potentially to dietary exposures plays a key role in the onset of NEC. This increased reactivity leads to an acute inflammatory response, intestinal cell apoptosis, mucosal destruction, and impaired mesenteric perfusion [75,76]. Intestinal mucosa recognizes bacterial products via pattern recognition receptors (PRRs) such as TLRs. The TLRs recognize microbial associated molecular patterns (MAMPs), which help regulate TLR expression [77]. Abnormal microbial colonization patterns or nutrient components can trigger inappropriate responses. The activation of specific TLRs leads to triggering nuclear factor kappa-beta (NF-κβ) and its inflammatory pathway propagating apoptosis [78,79]. Fatty acid composition and quantity modulates this inflammatory cascade via direct and indirect pathways (Figure 2).

### 6.1. Direct Effect of Fatty Acids on NEC Pathway

#### 6.1.1. TLR4

Several SFAs are shown to stimulate an inflammatory response through a TLR4 signaling pathway, while lower rates of NEC is associated with decreased TLR4 mRNA expression in the rats fed PUFAs [80,81,82]. A study by Lee et al. described the effect of different fatty acids on the TLR4 signaling pathway [82]. Saturated fats including lauric (C12:0), stearic (C16:0), and palmitic (C18:0) acids induced COX-2 expression through an NFκβ-dependent mechanism in a macrophage cell line [82]. Lauric acid showed the greatest activation capacity for TLR4 [82]. In contrast, MUFAs and PUFAs did not activate TLR4 signaling pathway. Of note, PUFAs (DHA; n3) and MUFAs (oleic acid; n9) significantly reduced the subsequent pro-inflammatory effect induced by lauric acid [82]. The degree of fatty acid unsaturation appears to have a key effect on TLR4 signaling. This study highlights the importance of keeping the balance between saturated and unsaturated fatty acids. SFAs are also an essential component of bacterial endotoxins that stimulate TLR4 [83]. The lipid A portion of lipopolysaccharide (LPS), a potent TLR4 ligand, has six SFAs with variable chain lengths between 12 to 16 carbons. Replacement of these SFAs by MUFAs or PUFAs is shown to stop the LPS pro-inflammatory activity [83]. Furthermore, the effect of PUFAs on the expression of TLR4 may not only influence the development of NEC, but also dictate its severity. In an animal model, Lu et al. revealed a negative association between PUFAs levels, the expression of TLR4, and the NEC severity [84].

#### 6.1.2. TLR2

Intestinal inflammatory response can also be induced by SFAs through the activation of TLR2; however, several conditions must be met to achieve this stimulation. Lee et al. reported the ability of lauric acid to induce activation through NF-κβ when TLR2 was co-transfected with TLR1 or TLR6, but not when TLR1, 2, 3, 5, 6, or 9 were individually transfected [85]. Of interest, DHA suppressed activation through the NF-κβ signaling pathway regardless of whether it was induced by lauric acid or LPS [81]. Similar findings were reported by Huang et al. who revealed that SFAs, particularly lauric acid, activate the NF-κβ signaling pathway in vitro for the inflammatory response through TLR2, when dimerized with TLR1 or TLR6, and TLR4 [86].

#### 6.1.3. Insulin Signaling Pathway

Other pathways are also described to explain the role that SFAs play in inducing inflammation. Pal et al. revealed the role of fetuin A, which acts as an endogenous ligand for TLR4, to promote lipid-induced insulin resistance [87]. Insulin, the only glucose-lowering hormone in the body, not only alleviates the detrimental effects of hyperglycemia, but also directly suppresses several TLRs, including TLR4, at the transcriptional mRNA levels [88]. Palmitic acid (C16:0) has been found to impair insulin signaling pathways by inducing insulin receptor substrate 1 phosphorylation, reducing the interaction between insulin and its receptors, and increasing the expression and secretion of pro-inflammatory cytokines [89,90,91]. 

#### 6.1.4. Platelet Activating Factor (PAF)

PAF has also been involved in inflammation, exaggerated apoptosis, and bowel necrosis in animal models and preterm infants [92]. Plasma PAF levels have been found to be significantly higher in neonates with NEC [93]. PAF causes apoptosis in enterocytes via a mechanism that involves Bax translocation to mitochondria, leading to collapse in its membrane potential, followed by subsequent activation of caspase 3 and DNA fragmentation [94]. Lu et al. indicated that PUFAs (AA and DHA), but not SFAs (palmitic acid), block this mechanism in ileal cells very early in the signaling cascade independently of any effect on prostaglandin synthesis, and likely directly via an effect on protein palmitoylation [95]. The use of LC-PUFAs may suppress the PAF pathway and partially alleviate the exaggerated inflammation associated with NEC. 

#### 6.1.5. Permeability

Regulated permeability is one of the dynamic functions of the enterocyte. The structure and function of intracellular tight junctions between enterocytes are vital to preventing translocation of commensal bacteria and maintaining the uptake of nutrients across the epithelial barrier. Under inflammatory conditions, enterocytes become inflamed, leading to increased permeability and chemical mediator production. The effects of PUFAs on permeability is complex and may be affected by the quantity and type of PUFAs [96]. EPA and DHA were shown to attenuate increases in permeability induced by pro-inflammatory cytokines and to prevent permeability changes induced by infection in a porcine epithelial cell model by preventing redistribution of tight junction proteins, claudin and ZO-1 [97,98]. Beguin et al. revealed limited effects of PUFAs on permeability and tight junction proteins in human intestinal epithelium under normal conditions [99]. In contrast, DHA limited the effect of the inflammatory stimulus on occludin, ZO-1, and barrier function during inflammation [99]. Similarly, Usami et al. reported that αLA, EPA, and DHA can increase permeability and decrease trans-epithelial electrical resistance when cytotoxic level of PUFAs are used [100,101]. The effects of PUFAs on intestinal permeability appear to be different between normal and inflammatory conditions. High doses of n3 PUFAs may increase intestinal permeability.

#### 6.1.6. T Lymphocytes

Infant formulas enriched with AA and DHA allow for enhanced T lymphocytes helper (Th1/Th2) response after T cell stimulation [102]. While this effect is described to support the establishment of oral tolerance [103], the role of the T helper response on the pathogenesis of NEC is yet to be determined. In mice models, TLR4 signaling in intestinal epithelial cells increased the migration of T cells to intestinal tissue and skewed the population of these cells in favor of pro-inflammatory type 17 T helper (Th17) cells over anti-inflammatory regulatory T (T_REG_) cells, leading to necrosis of intestinal tissue [104]. Of note, treatment with enteral retinoic acid, a metabolite of vitamin A, led to the restoration of the former balance towards T_REG_ cells and reduced the severity of NEC disease in these mice [104]. Intestinal retinoic acid metabolism is intensely affected by SFAs [105]. Retinoic acid supplemented with a high-fat diet is shown to accelerate the attenuation of intestinal adaptability and promote fat absorption gene expression in mice [106,107]. In a systematic review that included six RCTs [108], vitamin A supplementation in preterm infants was not shown to decrease the risk of NEC; however, none of the RCTs included in the systematic review examined the association between vitamin A and NEC in relation to fatty acid type or amount. The interaction with vitamin A needs to be considered in future studies examining the benefits of LC-PUFAs supplementation.

### 6.2. Indirect Effect of Fatty Acids on the Pathogenesis of Necrotizing Enterocolitis

#### 6.2.1. Intestinal Microbiome

Intestinal microbiome dysbiosis plays a central role in the development of NEC [109]. Several studies have revealed that the intestinal microbiome of preterm infants with NEC is profoundly different from the microbiome of those who are unaffected by NEC [110,111,112]. Nevertheless, there are multiple organisms implicated in these studies that highlight the complexity of the intestinal microbiome and the lack of a single causative agent. A predominance of Proteobacteria and a marked reduction in *Firmicutes* have been frequently described in fecal samples from infants who developed NEC compared with control infants [110,111,112]. Breastmilk is a protective factor against developing NEC by modulating the gut microbiome towards a *Bifidobacterium*-rich community. While this effect is mainly attributed to the human milk oligosaccharides (HMOs), breastmilk is also the main source of fatty acids for preterm infants, which could provide another mechanism of modulating the gut microbiome [113]. Additionally, breastmilk microbiota, as one potential source of microbes to the infant’s gut, are affected by the fatty acid content of breastmilk [113,114]. How fatty acids in breastmilk alter the gut microbiome appears to be complex and remains largely unknown. Table 1 summarizes the studies that reported the effect of different fatty acid profiles on intestinal microbiota. It is noteworthy that the influence of dietary fat on the microbiome composition considerably varies between individuals in in vitro experimental models and expectedly even more so in infants. 

#### 6.2.2. Antibacterial Effects of Fatty Acids and Interaction with Probiotics

**Figure 3:**
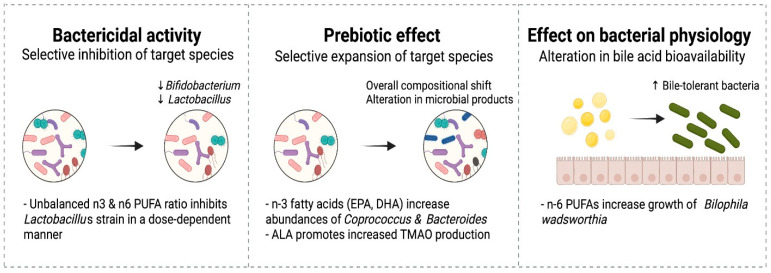
Effect of dietary fatty acids on the gut microbiome.

The bactericidal activity of several PUFAs has been long recognized and could compromise the actions of some native intestinal microbes and even supplemented probiotics [129,130]. Therefore, the potential inhibitory impact of some fatty acids on these species has important implications, particularly when probiotics are provided (Figure 3). Probiotics, mostly comprising *Bifidobacterium* and *Lactobacillus* species, are frequently used to promote healthy intestinal microbiome development and prevent NEC in preterm infants [131]. The effects of PUFAs on intestinal microbial species are dose-dependent. Kankaanpaa et al. examined these effects in vitro. Three *Lactobacillus* strains (GG, bulgaricus, and casei Shirota) were exposed individually to increasing concentrations of linoleic, γ-linolenic, arachidonic, α-linolenic and docosahexaenoic acids at physiological concentrations in growth media [132]. Higher concentrations of PUFAs inhibited growth and mucus adhesion at different threshold concentrations that varied by strain. However, the viability of the bacteria was not compromised by any PUFAs at any concentration. Arachidonic and γ-linolenic acids at low doses supported growth compared with controls. Whether similar exposures with stepwise changes in PUFA concentrations result in similar effects on microbial species in infants is yet to be studied.

#### 6.2.3. Prebiotic Effects of Fatty Acids

Prebiotics are increasingly recognized as a potential measure to promote intestinal health and prevent NEC in preterm infants [120]. While the term prebiotics has long been used to describe some nutrients that remain undigested, the definition has been recently revised to include ingredients that allow specific changes to the activity of the intestinal microbiome such as branched-chain fatty acids (BCFAs) and short chain fatty acids (SCFAs) [133]. n3 PUFAs, particularly EPA and DHA, have been described to increase production of BCFAs and SCFAs [116,134]. In a recent study, supplementation with n3 fatty acids containing EPA and DHA resulted in a significant increase in *Coprococcus* and *Bacteroides* species, and increased levels of certain gut-derived metabolites including BCFAs and SCFAs with similar effects to that seen with inulin, a well characterized prebiotic [116]. 

#### 6.2.4. Effects of Fatty Acids on Microbial Physiology

Fatty acids could impact the microbiome composition through their ability to promote the growth of bile-tolerant bacteria such as *Bilophila* [96,135,136]. A high saturated fat milk diet containing n6 LC-PUFAs helps the growth of *Bilophila wadsworthia*, a gram-negative bacillus that induces inflammation, intestinal barrier dysfunction, and bile acid dysmetabolism [135]. Accumulation of intestinal hydrophobic bile salts is implicated in the development of NEC in preterm infants [137]. By contrast, *B. wadsworthia* is not recognized when high n3 PUFA-based diet is used [135]. Thus, n3 LC-PUFAs supplementation may promote the growth of intestinal microbes that improve bile acid metabolism and reduce inflammation. 

#### 6.2.5. High Fat Diet and Intestinal Fat Overflow

High bioavailability of intestinal fat augments its impact on the microbiota. In a mouse model, a high fat diet consisting of palm oil SFAs resulted in decreased fat absorption, increased fecal fat overflow, and increased SFA transport [124]. These changes were associated with increased abundance of *Firmicutes* members bacilli and clostridia, and decreased microbiome diversity [124]. Preterm infants have low fat absorption capacity [138], and therefore are prone to fat “overflow” that may result in similar microbial changes. Furthermore, both standard and targeted fortification practices are shown to increase the fat content of fortified milk above the recommended intake [39]. To date, there are limited data on how much fat is too much for preterm infants on enteral feeding. In contrast, several studies examined the maximum protein allowed in this population [139].

#### 6.2.6. Interactions with Other Nutrients

The interactions between fatty acids and microbiota can also affect the function of other nutrients that are vital for intestinal vascular health. For example, choline is essential for fat absorption and provides substrate for phosphatidylcholine, a structural molecule in cellular membranes that is crucial for cell growth and function [140]. Choline is also the major nutrient precursor of gut microbe-dependent trimethylamine N-oxide (TMAO) [141], a metabolite that is shown to promote inflammation in vascular smooth muscle cells and aortic endothelial cells through mitogen-activated protein kinase and nuclear factor-κβ activations [142]. Although not fully understood, some fatty acids could impact TMAO production and endocannabinoid signaling [143,144]. For example, αLA is linked with microbiome compositional shifts resulting in lower production of TMAO [143]. αLA could potentially be used as a dietary supplement for preterm infants who have high levels of TMAO, theoretically predisposing them to higher risk of inflammation in the intestinal vascular system and also cardiovascular disease later in life [145].

## 7. Supplementation of LC-PUFAs and Risk of Necrotizing Enterocolitis

### 7.1. Parenteral Lipids

During the initial postnatal period, preterm infants often depend largely on parenteral nutrition. Parenteral lipid composition is therefore important to reduce postnatal deficiencies of LC-PUFAs. Until recently, Intralipid^©^ (Fresenius Kabi USA, Melrose Park, IL, USA) was the only available IVLE in North America. Intralipid^©^, made mainly of soybean oil, contains high amounts of n6 LC-PUFA and low amounts of n3 LC-PUFA with a 7:1 ratio. This results in prostaglandin synthesis favoring pro-inflammatory products and amplified oxidative stress. The ideal ratio of n6:n3 LC-PUFA for immune modulation is between 1:1 and 4:1 [146]. The concern about an unbalanced n6:n3 ratio has led to the development of products enriched with n3 fatty acids from fish oil, SMOFlipid^©^ (Fresenius Kabi USA, Melrose Park, IL, USA). The combination of soybean oil and fish oil delivers a balanced LC-PUFAs n6:n3 ratio of 2.5:1 and provides sufficient amounts of DHA and EPA. 

Although most preterm infants develop NEC when they are exclusively on enteral feeding, current evidence suggests that IVLEs may result in a unique fatty acid profile in the intestinal lumen. A recent study randomized preterm pigs to receive one of three IVLEs containing 100% soybean oil, 15% fish oil, or 100% fish oil with enteral feedings over an 8-day protocol [147]. These different IVLEs induced unique ileal fatty acid and metabolomic profiles with significantly higher DHA and EPA in the groups that received 15% or 100% fish oil; however, the incidence of NEC was similar between the three groups [147]. The results of this study suggest that short exposure durations of IVLEs are unlikely to mediate a change in risk of NEC. Whether a longer exposure of one of these IVLEs could influence the risk of NEC requires further investigation. Other studies on preterm pigs indicated that NEC develops mainly after enteral feeding and more often after proceeding parenteral nutrition of 2–3 days [148]. However, exposure to IVLEs, whether they contain fish oil or not, for shorter than 9 days did not alter the risk of NEC [147]. Current evidence suggests that early enteral feeding with colostrum in preterm piglets on soybean-based IVLEs decreases hepatic n6 LC-PUFA levels and leads to a higher n3 to n6 ratio but without altering the risk of NEC of the short term [149]. These studies indicate that early enteral feeding in preterm infants is important factor to prevent NEC regardless of the type of IVLEs. 

Clinical studies that compared n3-enriched IVLEs in preterm infants have small sample sizes and lack the power to detect NEC. A Cochrane review looking at the effect of fish oil-enriched IVLEs on the risk of BPD found only one RCT that included 59 preterm infants and showed no effect on the rate of NEC [150]. Another systematic review and meta-analysis included 386 preterm infants and found no association between type of IVLE and NEC [151]. 

### 7.2. Enteral Supplementation

Following the weaning of parenteral lipid, preterm infants rely on enteral sources to meet their LC-PUFA requirements, whether through breastmilk and human milk fortifiers, or formula. Among LC-PUFAs, DHA has received the most attention due to its important role in brain development. The DHA content in breastmilk of lactating mothers varies in direct correlation with their DHA intake. Current recommendations for DHA intake propose that pregnant and lactating women should have a minimum intake of 200–300 mg of DHA per day [152,153]. The worldwide mean DHA concentration of breastmilk is 0.32% [154]. Lower average concentrations are found in the breastmilk of lactating women on a typical Western diet (0.20%) and in donor human milk (0.10%) [143,155,156]. Supplementing breastmilk donors with 1000 mg of DHA daily has shown to increase the absolute milk concentration of DHA in donor human milk by 4 times [157]. Liquid human milk fortifiers, having approximately 0.15% DHA level, can also increase the DHA content of breastmilk to 0.29% of total fatty acids [158]. 

Additional supplementation of LC-PUFAs to preterm infants, either directly or indirectly through their lactating mothers, has been a target of several studies. Earlier studies explored the effect of DHA on long-term neurodevelopmental outcomes, while recent studies have focused on the potential benefits on neonatal morbidities such as BPD, ROP and NEC. LC-PUFA supplementation in these studies was achieved by adding fish oil, oil extracted from the fungus *Mortierella alpina* (AA), oil from the algae *Crypthecodinium cohnii* (DHA), and phospholipids (lecithin/phosphatidylcholine) from egg yolk [40,41]. The results of these studies showed limited effect of supplementation on the incidence of NEC except when the ratio between DHA and AA is maintained in the favorable range. Table 2 summarizes the RCTs that reported the effect of supplementation with different types and sources of LC-PUFAs on the incidence of NEC. All of the studies except Bernabe-Gracia et al. and Carlson et al. focused on other neonatal morbidities or long-term neurodevelopmental outcomes. LC-PUFAs supplementation was given either to the lactating mothers or directly to their preterm infants. Of the 10 studies that supplemented DHA alone, only one indicated a lower incidence of NEC. Six studies supplemented DHA and AA and showed a small influence on the incidence of NEC. It is worth noting that all six studies had small sample sizes and were unable to detect a significant difference in the incidence of NEC except one by Carlson et al. in 1998. This study was the only one that used egg phospholipid to provide DHA and AA.

## 8. Other Fat Supplementation

### 8.1. Branched-Chain Fatty Acids

BCFAs are predominantly SFAs and include mono-, di-, or poly-methyl BCFAs with ≥1 methyl branching point near the terminal end of the carbon chain. BCFAs are first introduced to the gastrointestinal tract through the ingestion of amniotic fluid comprising vernix caseosa particles [173,174]. After birth, BCFAs are provided to the infant through breastmilk. BCFA concentrations in breastmilk vary widely and are influenced by diet [175]. The impact of BCFAs on preterm infant health is not yet examined. Nevertheless, BCFAs have been reported to reduce the incidence of NEC in a neonatal rat model by over 56% [176]. In a study by Ran-Ressler et al., feeding rat pups formula containing 20% fat as BCFAs, similar to the concentration in vernix, resulted in significant shifts in ileal microbiota toward organisms that use BCFAs such as Bacillus subtilis and Pseudomonas aeruginosa [176]. The abundance of these organisms may mediate in the effect of BCFAs on the incidence of NEC [176]. Furthermore, pups who received BCFAs had higher pro-inflammatory IL-10 levels. A selective incorporation of these fatty acids into membrane phospholipids may be also associated with a protective effect of BCFAs against NEC [176].

### 8.2. Milk Fat Globule Membrane

Milk fat globule membrane (MFGM) is a unique triple-membrane structure produced during the process of fat secretion from the mammary gland epithelium. MFGM is the sole source of phospholipids in breastmilk and primarily consists of polar lipids, specific membrane-bound glycoproteins, and enzymes [177]. These bioactive molecules are largely absent from infant milk formula although the recent development of dairy technology has made it possible to extract MFGM from fresh bovine milk. MFGM exerts several beneficial effects on the gastrointestinal tract via its anti-inflammatory and anti-infective potency [178]. MFGM could reduce the severity of NEC by preserving mucosal integrity, reducing intestinal permeability, and attenuating oxidative stress [179,180,181,182,183]. In a study, MFGM supplementation in neonatal rats with NEC downregulated intestinal inflammation by inhibiting the expression of TLR4, myeloid differentiation primary response gene 88 (MyD88), and phosphorylated NF-κβ pathway [181]. Another recent study revealed that enteral MFGM supplementation can alleviate colonic barrier dysfunction in a rat model of short bowel syndrome, possibly via strengthening the colonic mucus barrier and regulation of NOD-like receptor family pyrin domain containing 6 inflammasome [184]. In addition, MFGM supplementation is shown to protect against *Clostridioides difficile*-induced colitis by increasing the abundance of *Firmicutes phyla* and anti-inflammatory bacteria, including *Lactobacillaceae*, *Erysipelotrichaceae*, and *Lachnospiraceae* [179,184]. In infants born at term, supplementation with MFGM in the first 4 months of life led to fewer episodes of diarrhea and fever compared with those fed standard formula [185]. To date, only one small study examined the effects of MFGM in preterm infants [126]. The administration of sphingomyelin-fortified milk in this pilot randomized control trial of 24 VLBW infants was associated with improved neurobehavioral development scores on Bayley Scales of Infant Development II. There were no cases of NEC in the trial [126]. Future studies should focus on evaluating the potential preventive effects and therapeutic dosage of MFGM in preterm infants.

## 9. Conclusions

Fatty acids are critical for the intestinal health of preterm infants and have the potential to influence the risk of NEC. Several mechanistic pathways support the role of different fatty acids, not only in the pathogenesis, but also in the prevention of NEC. Despite the wide inter- and intra-individual variations of fat content of breastmilk, and the well-recognized differences between the fatty acids of breastmilk and infant formula, alterations of this important macronutrient to prevent NEC is seldom explored. These alterations could be achieved by modifying the diets of the lactating mothers for breast-fed infants, or by changing the source of fatty acids used in manufacturing formula for formula-fed infants. The addition of LC-PUFAs with an appropriate n3 to n6 ratio, TGs structured as sn2-palmatic acid, or MFGM are promising factors that may reduce intestinal inflammation and potentially decrease the risk of NEC in preterm infants. Future studies should focus on supplementing LC-PUFAs with a favorable n3 to n6 ratio and examine the influence of the source of the fatty acids on the incidence of NEC. Furthermore, the use of hydrolyzed fat in preterm infant formula and HMF warrants more clinical research. The design of formulas and HMFs should prioritize the types and levels of fats that will ultimately result in short- and long-term benefits for preterm neonates.

## Figures and Tables

**Table 1:** Effects of dietary fatty acids on intestinal microbiome in human and animal studies.

Author	Model	Fat Intake and Type	Microbiome Changes	Metabolomics Changes
Human studies—Infants				
Younge et al. [115] 2017	Randomized, controlled trial in preterm infants with enterostomy due NEC or SIP	Fish oil or safflower oil compared to standard of care - The fish oil supplements (EPA, DHA, and Vit E) - Safflower oil (enriched in n6 LA) for a goal n6 to n3 fatty acid ratio of 3.75 to 5.1	↓ *Proteobacteria* ↓ *Enterobacteriaceae* ↑ Actinobacteria ↑ *Enterococcus*	-
Human studies—Adults				
Vijay et al. [116] 2021	6-week randomized dietary intervention	n3 fatty acid supplementation - Daily supplementation with 500 mg of n3 (165 mg of EPA and 110 mg DHA)	↑ *Coprococcus* spp. ↑ *Bacteroides* spp. ↓ *Collinsella* spp. ↓ *Ruminococcus*	↑ Butyrate, iso-butyrate, isovalerate ↑ Total plasma n3 fatty acids
Watson et al. [3] 2018	8-week randomized, open-label, cross-over trial with 12-week washout	n3 PUFA supplements 2000 mg EPA and 2000 mg DHA per day in two formulations Four soft-gel capsules Smartfish Remune drinks	↑ *Bifidobacterium* ↑ *Roseburia* ↑ *Lactobacillus* *↓ Faecalibacterium*	-
Kjolbaek et al. [117] 2020	Cross-over design with two diet periods of 4 weeks with 4-week washout period	Arabinoxylan oligosaccharides and PUFA cross-over Daily PUFA intake of approximately 10% increasing the intake of PUFA including n3 fatty acids and lowering SFA intake. fish oil capsules containing 3.6 g/d n3 PUFA (DHA and EPA)	No change	-
Fava et al. [118] 2013	Randomized trial in adult volunteers with at least two features of metabolic syndrome	Participants followed a 4-week reference diet and then were randomly assigned to the intervention arms for 24 weeks. Reference diet: high SFA diet/high glycemic index Intervention arm: - High MUFA/high glycemic index - High MUFA/low glycemic index - High carbohydrate/high glycemic index - High carbohydrate/low glycemic index	↓ Total bacteria in high MUFA groups ↓ Total bacteria in high SFA compared to baseline ↑ *Faecalibacterium prausnitzii* in high SFA compared to baseline	↑ Acetate, propionate, and n-butyrate in high SFA compared to baseline
Wan et al. [119] 2019	6-month randomized controlled-feeding Trial in healthy young adults with normal BMI	The three isocaloric diets: - low-fat diet (fat 20% energy) - moderate-fat diet (fat 30% energy) - high-fat diet (fat 40% energy)	Low-fat diet: ↑ Shannon diversity ↑ *Blautia* ↑ *Faecalibacterium* Moderate-fat diet: ↑ *Bacteroidetes* High-fat diet: ↓ *Firmicutes* ↑ *Bacteroidetes* ↓ *Faecalibacterium* ↑ *Bacteroides*	-
Pig				
Che et al. [120] 2019	Piglets with intrauterine growth retardation	Diet contained either flaxseed oil (enriched in n3 PUFAs) compared to soy oil (high in n6)	↑ *Actinobacteria* ↑ *Melainabacteria* ↑ *Bifidobacterium* ↑ *Blautia* ↓ *Spirochaetes*	↓ Diarrhea ↑ villus height ↑ Ileal Claudin-1 and ZO-1 ↓ Ileal MyD88, NF-κB, TNF-α and IL-10
Anderson et al. [121] 2011	Piglet	Piglets were grouped into these treatments: - Fish oil (n3 LC-PUFA, providing 34% EPA and DHA in a 1:1 ratio) - Sunflower oil (n6 PUFA, 67% linoleic acid)	Fish oil diet: ↑ *Proteobacteria* ↑ *Actinobacteria* Sunflower oil diet: ↑ *Bacteroides* spp.	-
Mouse				
Liu et al. [122] 2012	Adult mice	Regular rodent chow for 14 days, and then mice received one of the three treatment groups for 10.5 weeks - High SFAs (soybean oil and fully hydrogenated soybean oil) - High n3 PUFAs (flaxseed oil, principally αLA, small amounts of EPA and DHA). - High n6 PUFAs (soybean oil principally LA)	↓ *Bacteroidetes*-to-*Firmicutes* in all groups ↓ *Bacteroidetes* in all groups (more in SFA-rich group) ↓ *Porphyromonadaceae* in n6 PUFA-rich group ↓ *Lachnospiraceae* in SFA-rich group	-
Ghosh et al. [123] 2013	Adult mice	Mice were weaned onto two high-fat diets fed for 5 weeks. - High n6 PUFA (corn oil) - High n6 and n3 PUFA (corn oil and fish oil containing 0.5–1.8 g of EPA and DHA	High n6 PUFA: ↑ Enterobacteriaceae ↑ *Clostridia* spp. High n3 PUFA: ↑ *Bifidobacteria* ↑ *Lactobacillus* ↑ *Enterococcus faecium*	-
de Wit et al. [124] 2012	Adult mice	Standard chow for 3 weeks followed by a low-fat diet based on palm oil for 3 weeks. Then either maintained on the low-fat diet or received high-fat diets for 8 weeks on Palm oil, Olive oil, or Safflower oil	↑ *Firmicutes* members bacilli and clostridia ↓ microbiome diversity	↑ Fecal fat overflow (more in Palm oil diet) ↓ Fat absorption ↑ Intestinal SFA transport
Saeedi Saravi et al. [125] 2020	Old mice	Standard chow until 8–12 weeks of age. One group remained on standard chow and the other mice received modified diets until >18 months of age. - High αLA (7.3%) - Low αLA (0.03%)	High αLA diet: ↓ decreased Faith’s phylogenetic richness ↓ *Ruminococcaceae* ↓ *Clostridiaceae* ↓ *Lachnoclostridium* ↑ *Bilophila*	↑ Acetate ↓ Trimethylamine N-oxide
Marques et al. [126] 2015	Adult mice	The animals were divided into two groups and received the intervention for 8 weeks. - Standard diet - Standard diet supplemented with 0.5% trans-10, cis-12 conjugated linoleic acid	↓ *Firmicutes* ↓ *Bacteroidetes* ↓ *Desulfovibrionaceae* ↓ *Peptococcaceae* ↑ *Porphyromonadaceae*	↑ Acetate ↑ Propionate ↑ Isobutyrate
Ghezzal et al. [127] 2020	Adult mice	Three-month-old male were fed standard chow diet. High fat mice received with palm oil rich in saturated palmitic acid (about 45%) and unsaturated oleic acid (about 35%)	↓ *Clostridium leptum* ↓ *Akkermansia muciniphila* ↑ *Bacteroides*	↑ Intestinal permeability
Huang et al. [128] 2013	Adult male mice	Intervention arms included isocaloric high-fat diets, where the dietary fat consisted of: - Milk fat - Lard - Safflower oil (rich in PUFA)	↓ *Bacteroides* in all groups compared to low fat control ↑ *Proteobacteria* in milk fat and PUFA groups	-

↑ increase; ↓ decrease; SIP, spontaneous intestinal perforation; NEC, necrotizing enterocolitis; EPA, eicosapentaenoic acid; DHA, docosahexaenoic acid; Vit E, vitamin E; PUFA, polyunsaturated fatty acid; MUFA, monounsaturated fatty acid; SFA, saturated fatty acid; ZO-1, zona occludens-1; MyD88, myeloid differentiation primary response 88; NF-κB, nuclear factor kappa B; TNF-α, tumor necrosis factor alpha; IL-10, interleukin 10.

**Table 2:** Clinical trials of effect of enteral DHA on the risk of necrotizing enterocolitis in human studies.

Author & Year	Population	No. of Participants	Supp. Intervention	Supp. Control	n3:n6 Balance	Feeding Type	Start of Intervention	Duration of Supp.	Dose	Primary Outcome	Definition of NEC	Incidence of NEC
Bernabe-García et al. [159] 2021	1000–1500 g	DHA: 100 Control: 100	DHA	High-oleic sunflower oil (MUFA)	No	Human milk or enteral formula	1st feed after birth	14 days	DHA 75 mg/kg/day	NEC stage ≥ IIa	Modified Bell’s criteria	DHA: 0% Control: 7%; *p* = 0.007
Marc et al. [160] 2020	23–28 weeks gestation	Intervention: 273 Control: 255	oral capsules of DHA given to mothers	Placebo capsules given to mothers	No	Breast milk	within 72 h of delivery	Until 36 weeks CA	DHA 1.2 g/day of for the intervention group	BPD-free survival	Modified Bell’s criteria	Placebo: 3.0% DHA group: 5.4% *p* = 0.14
Collins et al. [161] 2017	<29 weeks gestation	Intervention: 631 Control: 642	DHA from fish oil	Placebo from soy without DHA	No	Breast milk or formula	within 3 days after their first enteral feeding	Until 36 weeks CA	DHA 60 mg/kg/day	BPD	Proven NEC	Intervention: 8.3% Control: 7.1% *p* = 0.46
Baack et al. [162] 2016	24–34 weeks gestation	Intervention: 31 Control: 29	DHA liquid	Placebo	No	Breast milk or formula	First week of life	Until discharge or 37 weeks CA	DHA 50 mg/day of for the intervention group	Feasibility and biochemical efficacy	N/A	0% in both groups
Makrides et al. [163] 2009	<33 weeks gestation	High DHA: 322 Standard DHA: 335	High DHA: mothers taking tuna oil capsules at ~1% DHA (or high-DHA preterm formula (~1% DHA and 0.6% AA)	Standard DHA: mothers taking soy oil capsules at ~0.3% DHA (or standard preterm formula at ~0.35% DHA and 0.6% AA)	No	For mothers providing breastmilk to their infants	Within 2–4 days of life	Until term CA	High-DHA: six 500-mg tuna oil per day Standard-DHA: six 500-mg soy oil per day	ND at 18 months CA	Not specified	High DHA group: 4.3% Standard DHA group: 2.1% Adjusted *p* = 0.10
Henriksen et al. [164] 2008	<1500 g	Intervention: 68 Control: 73	Soy oil and MCT + AA and DHA as triacylglycerol	Soy oil and MCT without DHA or AA	Yes	Human milk (EBM/DHM)	1 week after birth	Until discharge (average, 9 weeks)	DHA: 32 mg and AA: 31 mg per 100 mL of human milk	ND at 6 months of age	Modified Bell’s criteria	Control: 2.7% Intervention: 1.5%
Groh-Wargo et al. [165] 2005	750–1800 g and <33 weeks gestation	Fish/Fungal oil: 20 Egg/Fish oil: 18 Control: 22	DHA + AA from fish/Fungal oil vs. DHA + AA from egg/fish oil	Regular with non-detected DHA/AA	Yes	Breastmilk or formula	From the first enteral formula feeding	Until 12 months CA	Fish/Fungal 24 kcal: DHA 0.27 g/100 g AA 0.43 g/100 g Egg/Fungal 24 kcal: DHA 0.24 g/100 g AA 0.41 g/100 g	Growth and body composition	Radiographic evidence NEC or surgical NEC	0% in all groups
Clandinin et al. [166] 2005	≤35 weeks gestation	Algal-DHA: 112 Fish-DHA: 130 Control: 119	Algal-DHA with AA from fungal oil vs. fish-DHA with AA from fungal oil	Regular with no DHA or AA	Yes	Formula	Average 30 + 5 to 31.2 wks CA	Until 92 weeks CA	Algal-DHA: 17 mg/100 kcal from algal oil and 34 mg ARA/100 kcal from fungal oil Fish-DHA: 17 mg DHA/100 kcal from fish oil and 34 mg ARA/100 kcal from fungal oil	Growth	Modified Bell’s criteria Stage II or III	Control: 2.5% Algal-DHA: 5.4% Fish-DHA: 5.4%
Fewtrell et al. [167] 2004	≤2000 g and <35 wks gestation	LC-PUFA: 122 Control: 116	LC-PUFA	Regular	Unclear	Formula	14 days	Until 9 months corrected age	AA: 0.4 g/100 g of fat DHA: 0.5 g/100 g of fat γ linolenic acid: 0.9 g/100 g of fat	ND at 18 months	Bell’s criteria or via surgery, or postmortem autopsy	Control: 1.7% LC-PUFA: 4.1%
Innis et al. [168] 2002	VLBW	DHA: 65 DHA + AA: 66 Control: 60	DHA at 0.34% vs. DHA at 0.33% and + AA at 0.60%	Regular with no DHA or AA	Yes in DHA + AA only	Formula	After reaching an enteral intake of 375 kJ/kg/d	At least 28 days	DHA formula: ~0.15% of energy as DHA DHA + AA formula: 0.14% and 0.27% of energy as DHA and AA, respectively	Growth	Suspected or confirmed	Control: 1.7% DHA: 3.1% DHA + AA: 0%
Fewtrell et al. [169] 2002	<1750 g and preterm	Control: 100 LC-PUFA: 95 Breastfed: 88	LC-PUFA from vegetable oils, milk fat, evening primrose oil and egg lipids	Preterm infant formula without additional LC-PUFA	Yes	Formula	5 ± 4 days after birth	Control: 33 ± 17 days LC-PUFA: 31 ± 21 days	AA 0.31 g/100 g of fat DHA 0.17 g/100 g of fat	ND at 18 months	Bell’s criteria or via surgery, or postmortem autopsy	Control: 2% LC-PUFA group: 5.3% *p* = 0.11
O’Connor et al. [170] 2001	750–1800 g	AA + DHA from Fish/Fungal oil: 140 AA + DHA from egg-TG/Fish oil: 143 Control: 144	AA + DHA (Fish/Fungal oil) vs. AA + DHA (egg-TG/Fish oil	Regular (non-detected DHA/AA)	Yes	Human milk or formula	within 72 h of the first feed	To 12 months CA	AA + DHA (Fish/Fungal oil) -In hospital: DHA 0.27 g/100 g total fatty acids, AA 0.43 g/100 g AA + DHA (Egg-TG/Fish oil) - In hospital: DHA 0.24 g/100 g AA 0.41 g/100 g	growth, visual acuity, and ND	Bell’s criteria or via surgery, or postmortem autopsy	Control: 4% AA + DHA (Fish/Fungal): 4% AA + DHA (Egg-TG/Fish): 3%
Carlson et al. [171] 1998	725–1375 g & ≤32 wks gestation	DHA + AA: 34 Control: 85	Egg phospholipid: DHA (0.13%) AA (0.41%)	Regular	Yes	Formula	At a mean of 4.9 days of age	After discharge home	7-fold more esterified choline, AA, and DHA in experimental formula	NEC stage II or III	Modified Bell’s criteria	DHA + AA: 2.9% Control: 17.6% *p* < 0.05
Carlson et al. [172] 1996	747–1275 g	DHA + EPA: 35 Control: 36	Marine-oil-supplemented formulas at 0.2% DHA & 0.06% EPA	Standard preterm formula	No	Preterm formula until 2 months CA	3–5 days of age	Until 48 ± 1 weeks CA	Standard preterm formula contained LA (1.2% of energy)	Visual acuity and growth	Not specified	DHA + EPA: 25.7% Control: 8.3%

NEC, necrotizing enterocolitis; EPA, eicosapentaenoic acid; DHA, docosahexaenoic acid; LA, linoleic acid; AA, arachidonic acid; LC-PUFA, long chain polyunsaturated fatty acid; MUFA, monounsaturated fatty acid; BPD, bronchopulmonary dysplasia; ND, neurodevelopment outcoem; MCT, medium chain triglyceride; CA, corrected age; EBM, expressed breastmilk; DHM, donor human milk.

## Data Availability

Not applicable.
